# Supplementary material for: Microevolution and genomic epidemiology of the diphtheria-causing zoonotic pathogen Corynebacterium ulcerans
Source: Nat Commun. 2025 May 24;16:4843. doi: 10.1038/s41467-025-60065-0 (PMC12103533; doi:10.1038/s41467-025-60065-0)
Supplement: Supplementary file 1 — Supplementary information [file 41467_2025_60065_MOESM1_ESM.pdf]

Supplementary material: Microevolution and genomic epidemiology of the diphtheria-causing zoonotic pathogen *Corynebacterium ulcerans*

Chiara Crestani[1], Virginie Passet[1], Martin Rethoret-Pasty[1], Nora Zidane[1], Sylvie Brémont[2], Edgar Badell[2], Alexis Criscuolo[3], Sylvain Brisse[1,2,3*]

[1] Institut Pasteur, Biodiversity and Epidemiology of Bacterial Pathogens, Paris, France

[2] Institut Pasteur, National Reference Center for Corynebacteria of the *diphtheriae* complex, Paris, France

[3] Institut Pasteur, Biological Resource Center of Institut Pasteur, Paris, France

Contents:

Supplementary text……………………………………………………………………………...p. 2-5

Supplementary figures S1-S11…………………………………………………………………..p.6-16

Supplementary table S1…………………………………………………………………………p. 17

Supplementary References…………..…………………………………………………………p. 18

Supplementary text

Definition of the cgMLST scheme

From a starting dataset of 347 genomes, we selected 239 representatives of the diversity of *C. ulcerans*. These included representatives of all ST (n=38) that had ≥21 nucleotide mismatches within the core genes.

The following functions were used, which are available within the chewBBACA suite:

i) whole genome multilocus sequence typing (wgMLST) with default settings (BLAST Score Ratio of 60%, CDS size variation threshold of 20%);

ii) allele call using the wgMLST scheme (paralogs are eliminated automatically at this step);

iii) evaluation of wgMLST call quality per genome;

iv) definition of the cgMLST scheme (95% threshold); the following user-defined options were implemented:

   a. a custom Prodigal training file (Culcerans_0102, the largest genome size and *tox* gene positive assembly available) was used to better identify CDS;

   b. removal or repeated loci;

   c. removal of genomes outside the ranges of 53.33% ± 0.51% % (corresponding to twice the standard deviation) for GC content and/or of 2,533,898 bp ± 180,868 bp (twice the standard deviation) for genome length.

This approach resulted in an initial cgMLST scheme of 1,941 loci, from which loci from both the seven-gene and the ribosomal MLST scheme[1,2] were discarded (total hits n=53), leading to 1,888 cgMLST loci.

These 1,888 loci were compared to the ones defining the cgMLST scheme for *C. diphtheriae* (n=1,305 loci) by BLASTn similarity searches (word size=10, e-value≤0.0001), using queries from the type strains of the two species (*C. ulcerans* NCTC7910^T and *C. diphtheriae* NCTC11397^T; Figure S8). Using 69% nucleotide identity and 70% query coverage thresholds (selected from the distributions of these two variables, Figure S9), and allowing 5% allele size variation, 519 loci were found to be in common between the two schemes (40% and 30% of the *C. diphtheriae* and *C. ulcerans* cgMLST schemes, respectively).

The expanded dataset (n=434; including 36 *C. ramonii* genome assemblies) was first tagged with the 1,888 cgMLST loci (with the `autotag.pl` script and the following parameters: -w 30, -f), then

scanned for new alleles (with the `scannew.pl` script and the following parameters: -w 30, -f, -c, 90% query coverage and 90% identity), and finally re-tagged to attribute novel alleles to the genomes. A total of 721,064 out of 819,392 maximum possible CDS (88%) were identified. The 251 loci showing ≥10% of missing data were discarded from the scheme, leading to 1,637 remaining loci.

To filter out loci with non-reproducible allele calls at low assembly coverage depths, alleles were called from *de novo* assemblies inferred using fq2dna on subsampled short reads (10×, 20×, 30×, 50×, 80×, 100×; three read subsamples for each coverage depth) from four isolates (FRC0027, FRC0058, FRC0687, FRC0804). No allele mismatches were detected at 80× and 100×, whereas nine loci showed non-reproducible allele calls at 30× and 50× (Figure S10). These nine loci discarded, leading to a final scheme based on 1,628 loci.

Implementation of the cgMLST scheme for *C. ulcerans* in the *Corynebacterium diphtheriae species* complex BIGSdb database

The 1,628 selected loci were ordered according to their position within the reference chromosome of *C. ulcerans* (strain 809, GCF_000215645.1). Loci shared with the cgMLST of *C. diphtheriae* were not renamed (n=497), whereas remaining loci (n=1,131) were named based on their position in the reference genome, incrementally (e.g., ULC_0001, ULC_0002, ...). Type alleles for *C. ulcerans,* (which are used at the scannew step to search for novel alleles) were registered for those loci (n=497) in common with the *C. diphtheriae* scheme. These were then incorporated in the *Corynebacterium diphtheriae* Species Complex (CdSC) BIGSdb database (https://bigsdb.pasteur.fr/diphtheria) as a dedicated scheme (cgMLST_ulcerans).

All genomes in the expanded dataset (n=434) were tagged on BIGSdb for 1,628 loci (`autotag.pl` script, see parameters above), and only those sequenced with Illumina technology (n=420) were used to define new alleles with the `scannew.pl` script; then, the whole set of genomes was tagged again, and cgMLST profiles were defined with the `define_profiles.pl` script, tolerating a threshold of 5% missing alleles (n≤81) to define cgSTs in the seqdef database. The same process was repeated to call alleles and define profiles in the additional isolates (n=235) of the third dataset.

Toxin gene and *tox*-associated mobile elements

Tox-prophages sequences were manually curated using Geneious Prime 2024.0. When *tox*-positive genomes were negative for the presence of either the PAI or prophages, manual exploration was carried out using SnapGene Viewer v7.1.0 (https://www.snapgene.com).

Definition and evaluation of a cgMLST scheme for genotyping of *C. ulcerans* and *C. ramonii*

We defined a set of 1,628 protein-coding loci for genotyping both *C. ulcerans* and *C. ramonii* using a sole cgMLST scheme. Locus lengths varied from 204 to 9,099 bp, and all concatenated loci had a total length of 1,682,922 bp (calculated using allele 1 for each locus), representing 69% of the chromosome length of the reference strain NCTC7910^T.

The allele call rate for *C. ulcerans* was defined based on 235 genome assemblies that were not used during the construction step of the cgMLST scheme, including genomes from Belgium (n=34), Germany (n=47), Japan (n=5), Norway (n=1), Spain (n=4), the UK (n=3), unknown geographical origin (n=2), and newly-sequenced genomes from the French NRC (n=139). The allele call rate was found to be 99.7% ± 0.2%, with 1,623 called alleles per genome on average. For the poorly sequenced *C. ramonii* strains, only four additional genome sequences (not involved for cgMLST building) were publicly available. Therefore, for scheme validation, we calculated the allele call rate for all *C. ramonii* isolates available (n=40, n=36 of which from the second dataset, excluding five non-Illumina-based genome sequences), showing an allele call rate of 96.5% ± 0.9% and an average of 1,571 called alleles per genome.

Based on the 582 genomes of the overall study dataset, each locus shares 2 to 91 unique alleles, with the number of distinct alleles increasing with locus size (Figure S11). The longest locus was atypically long (9,099 bp), coding for a type I polyketide synthase, and had the highest number of alleles as expected from the positive correlation between locus size and allele number.

Population structure of *C. ulcerans*

For continuity of the nomenclature, each clonal group (CG) and sublineage (SL) cluster (derived from single-linkage classification) was named by inheriting the identifier from the most common seven-gene Sequence Type (ST) in that cluster (Figure S2); when more than one CG uniquely or mostly comprised the same ST, incremental numbers starting at 10,000 were attributed to the smaller groups

(e.g., for two groups predominantly made of isolates with ST326, CG316 and CG10000 were attributed).

MLST data from the seven-gene scheme led to 54 different STs, with four very common ones: ST325 (n=179, 31%), ST331 (n=99, 17%), ST339 (n=62, 11%) and ST332 (n=51, 8.8%). These comprised 67.2% of the isolates in the validation dataset (unless otherwise stated, we use the validation dataset for epidemiological inferences hereafter). STs were not always concordant with the phylogenetic structure of *C. ulcerans* (Microreact project), as ST326, ST331 and ST332 were polyphyletic; however, all other non-singleton STs were monophyletic. Of note, ST325 was monophyletic but subdivided into four different clonal groups (CG325, CG583, CG543, CG10001; see below).

Other virulence genes

The phospholipase D (PLD, a sphingomyelinase) is another important virulence factor of *C. ulcerans*. We observed that 557 genomes (95.7%) carried the PLD exotoxin gene *pld*, with only SL349 being negative for this gene.

The *rbp* (ribosomal-binding protein) gene, another virulence factor described in the CdSC, was carried uniquely by one genome (809, an ST338 which is the reference genome for *C. ulcerans*).

Mobile genetic elements associated with the diphtheria toxin gene

For 36 genomes, it was not possible to clearly identify which genetic element was carrying the toxin, due to genome assembly fragmentation.

Regarding ILE integrases, ILE-1 and ILE-2 were identical except for their sequence length (150 AA and 104 AA, respectively), whereas integrase ILE-3 shows a lower amino acid sequence identity (82%). ILE-1, ILE-2 and the PAI integrases were more similar to phage integrases 1 and 2, than was phage integrase 3.

Supplementary figures

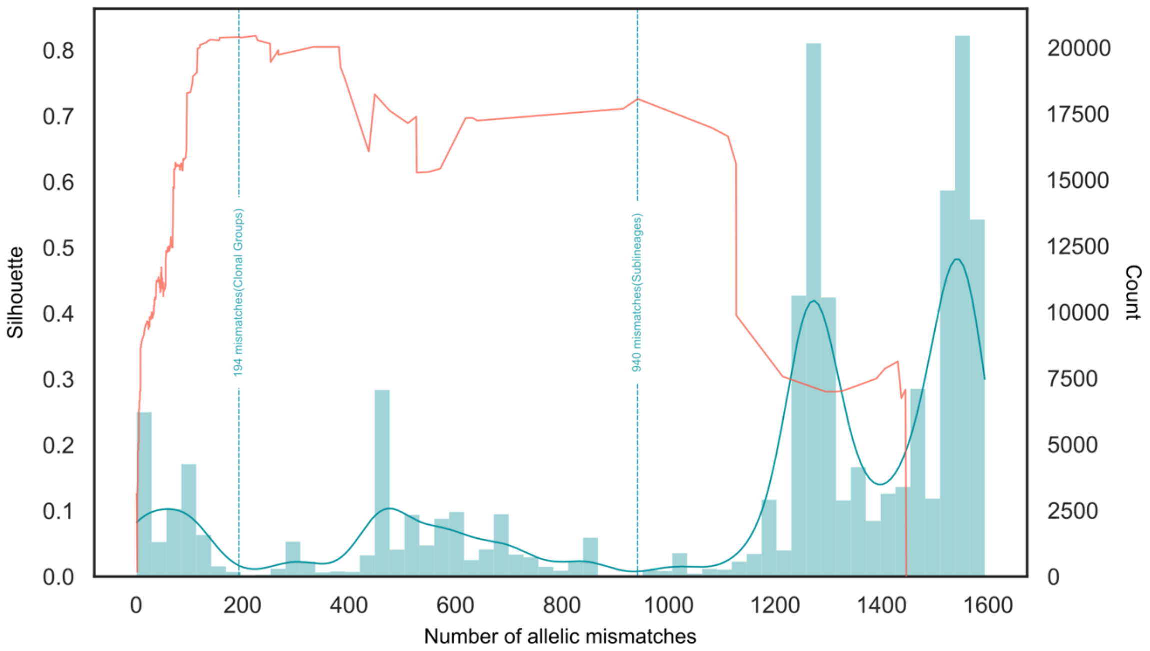

Figure S1. Frequency plot illustrating the number of cgMLST allelic mismatches between all pairs of *Corynebacterium ulcerans* genomes in the validation dataset (n=177), except for those with incomplete cgMLST profiles (n=5) and duplicated genomes (n=2). The silhouette coefficient (S_t) was plotted as a red line. The y-axis on the left shows silhouette values, while the one on the right shows the number of profile pairs. The two dotted lines correspond to the selected thresholds for clonal group (194 mismatches) and sublineage (940 mismatches) definitions.

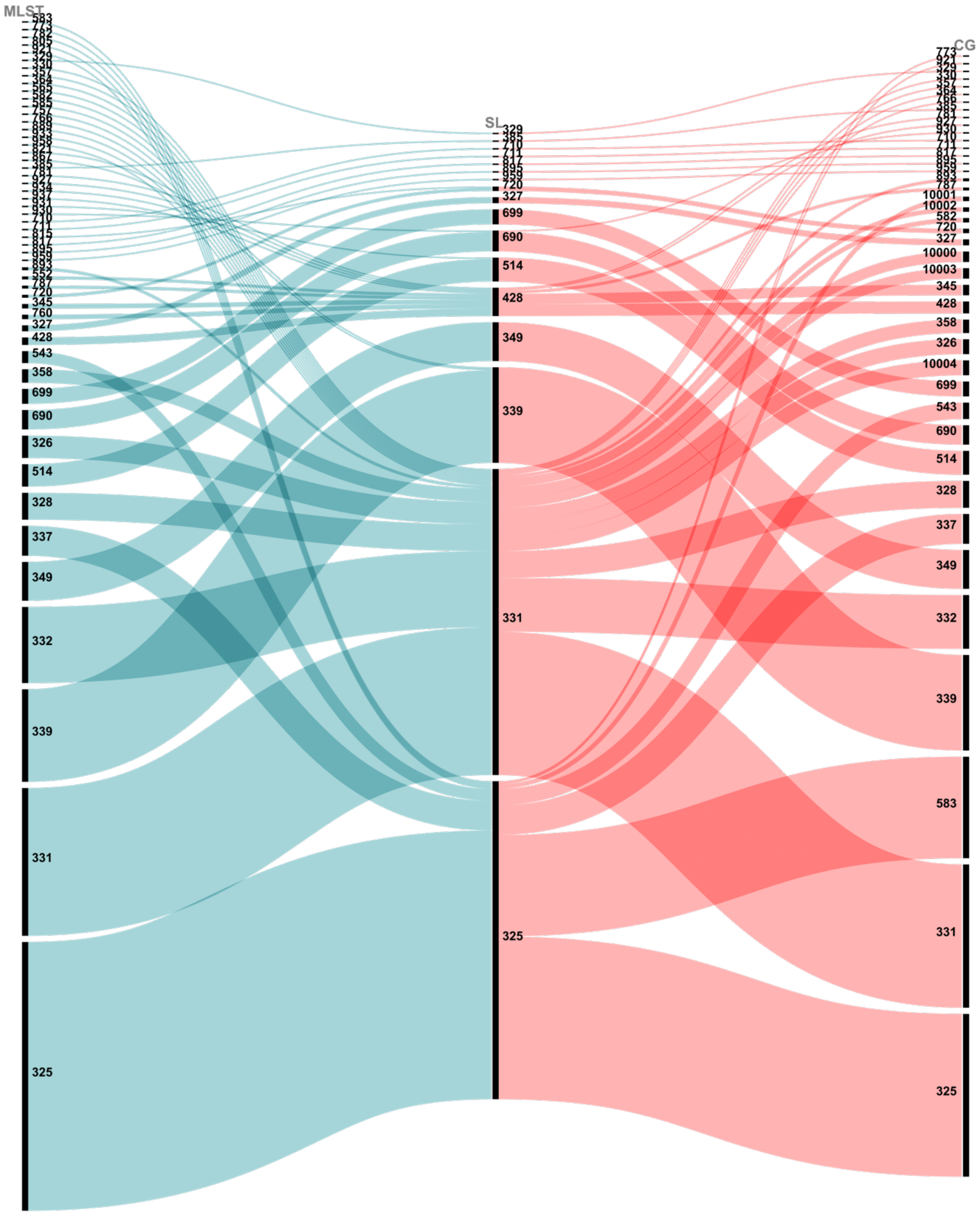

Figure S2. Alluvial diagram showing how *C. ulcerans* Sequence Types (ST – MLST) from n=577 genomes map to its sublineages (SL) and clonal groups (CG), and the inheritance of the seven-gene MLST nomenclature of SL and CG.

Reported clusters of *C. ulcerans* linked with cryptic clusters

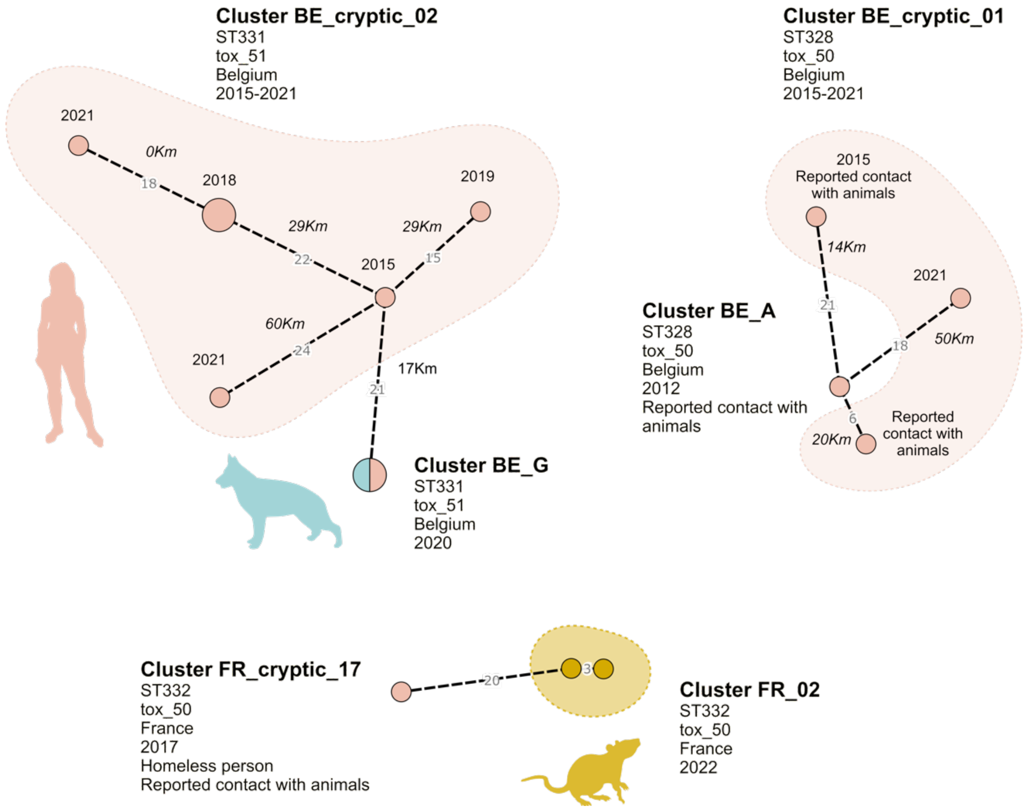

Figure S3. Three cryptic clusters linked to reported outbreak clusters detected in this work and identified with single-linkage clustering (threshold of 25 allelic mismatches). On the top left, cluster BE_cryptic_02 (n=6 genomes) is linked to cluster BE_G (n=2 genomes); on the top right cluster BE_cryptic_01 (n=3) and BE_A (n=1); and on the bottom center cluster FRC_cryptic_17 (n=1) and FR_02 (n=2).

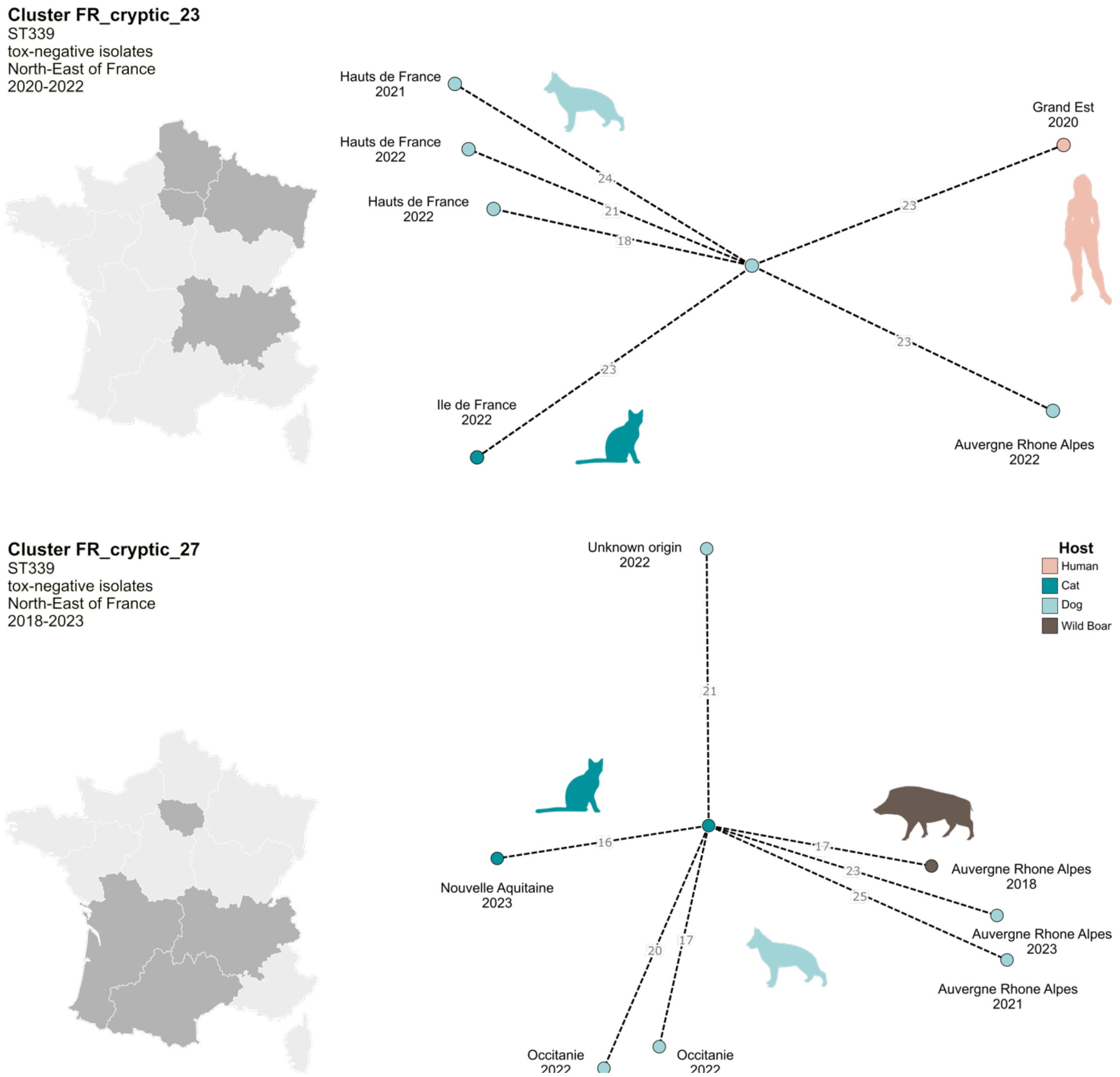

Figure S4. Two major cryptic clusters detected in this work, FR_cryptic_23 (n=7 genomes) and FR_cryptic_27 (n=8 genomes). These clusters show a strong association with distinct geographical areas.

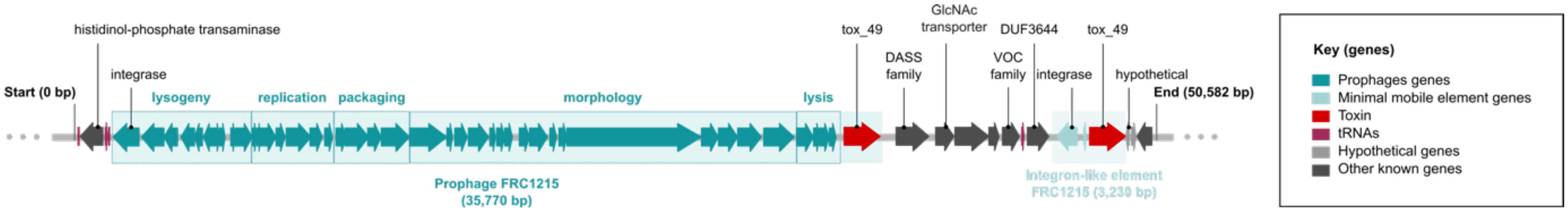

Figure S5. Map of a chromosomal region of the hybrid (short- and long-reads) assembly of the isolate FRC1215, shown here as representative of CG325 genomes (n=109), all of which carry an orphan toxin in their short-read-based assembly. Long-reads helped in elucidating both the cause of the incorrect short-read-based assemblies (i.e. a double copy of the toxin), and the mobile genetic elements carrying the toxin (a prophage and an integron-like-element).

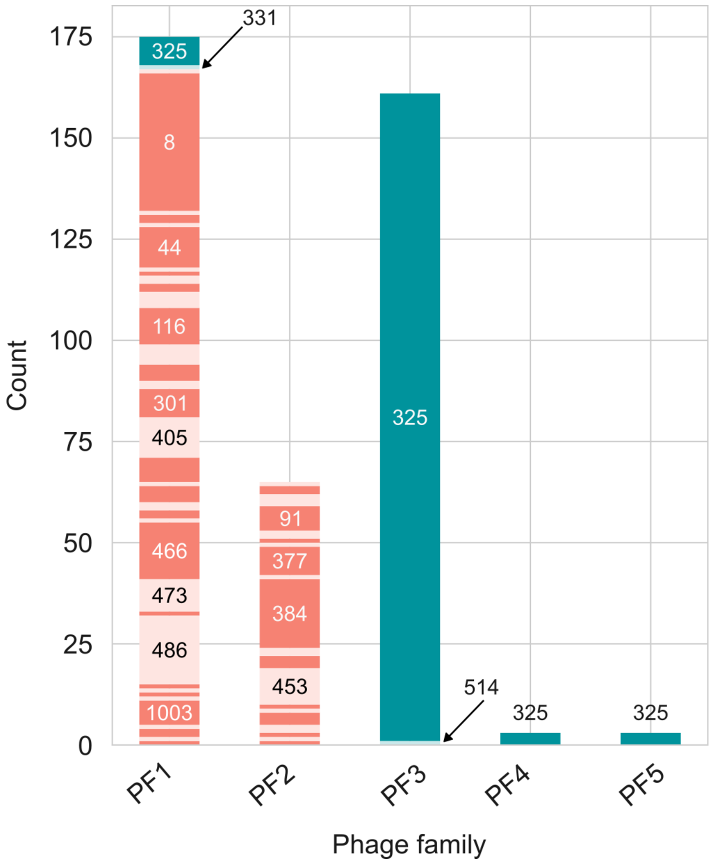

Figure S6. Bar chart showing the association of prophage families (PF, n=4) with sublineages (SL) of *C. diphtheriae* (shades of pink) and *C. ulcerans* (shades of blue). PF3, PF4 and PF5, which were only detected in *C. ulcerans* genomes, are uniquely associated with one lineage (SL325), except for one phage from PF3 (SL514), whereas PF1 and PF2 are associated with multiple phylogenetic lineages of *C. diphtheriae*, and in addition for PF1, with a few *C. ulcerans* that belong to SL325 and SL331.

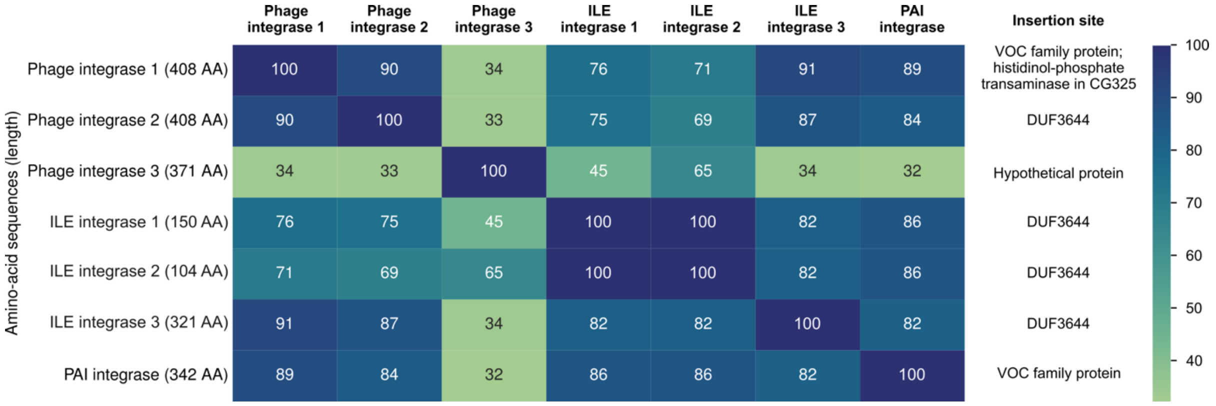

Figure S7. Heatmap of the pairwise amino-acid sequence identities of integrase genes belonging to *tox*-carrying mobile genetic elements (MGE) detected in this study. Insertion sites where each of these were detected are indicated in the last column; most integrases are site-specific (one insertion site only), except for phage integrase 1, which had a different insertion site in GC325 genomes. Some of these integrases share the same insertion site (e.g. phage integrase 2, with ILE integrase 1, 2 and 3; and phage integrase 1 with the PAI integrase), which likely explains why these elements appear mutually exclusive.

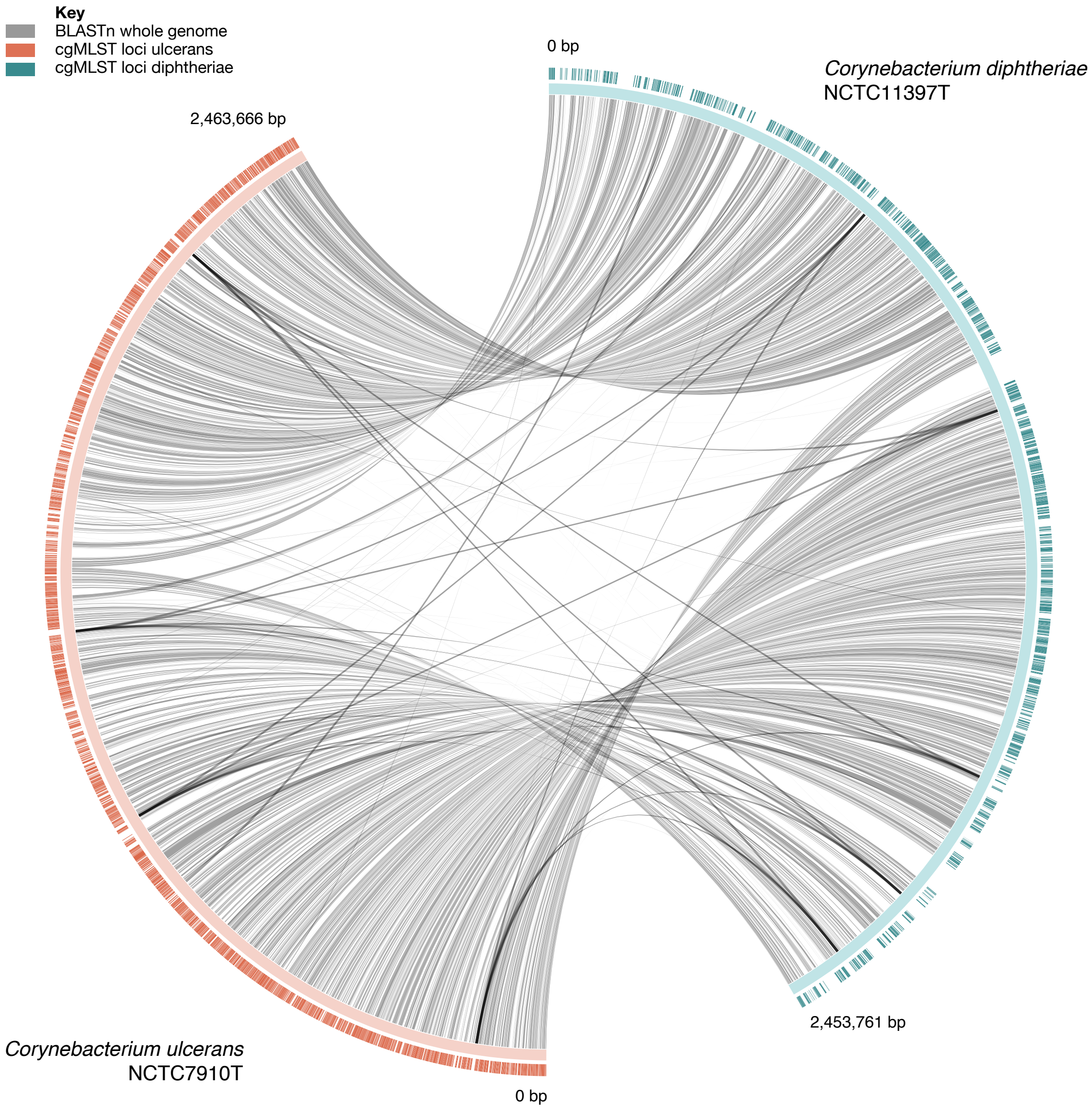

Figure S8. Circular plot representing the reference genomes *C. ulcerans* NCTC7910^{T} and *C. diphtheriae* NCTC11397^{T} (continuous orange and green lines, respectively), and the genome positions of their respective cgMLST loci. Alleles in common are represented by grey ribbons (indicating their genome identity, n=519; BLAST ID >69%, query coverage >70%). Black represents 100% identity; the lightest grey, 69%.

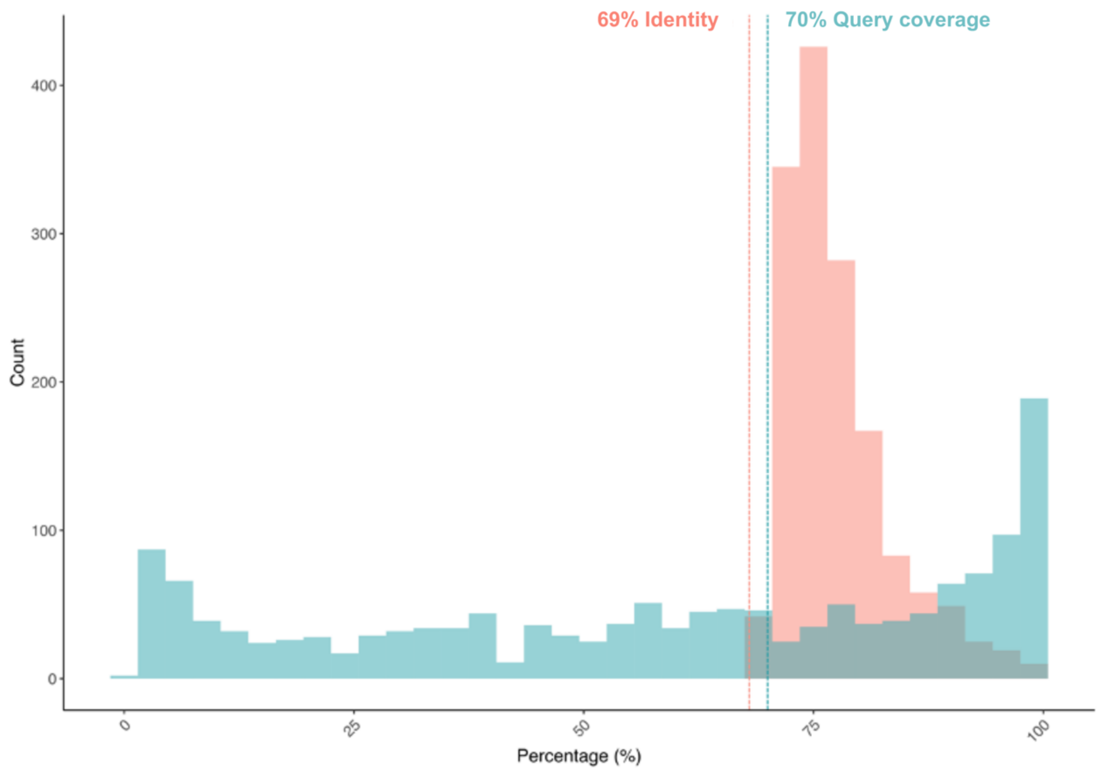

Figure S9. Distributions of the nucleotide identity (in pink) and of query coverage (in blue) values derived from pairwise blast alignments between the *C. diphtheriae* cgMLST loci (n=1,305) and the filtered *C. ulcerans* cgMLST loci from chewBBACA (n=1,888).

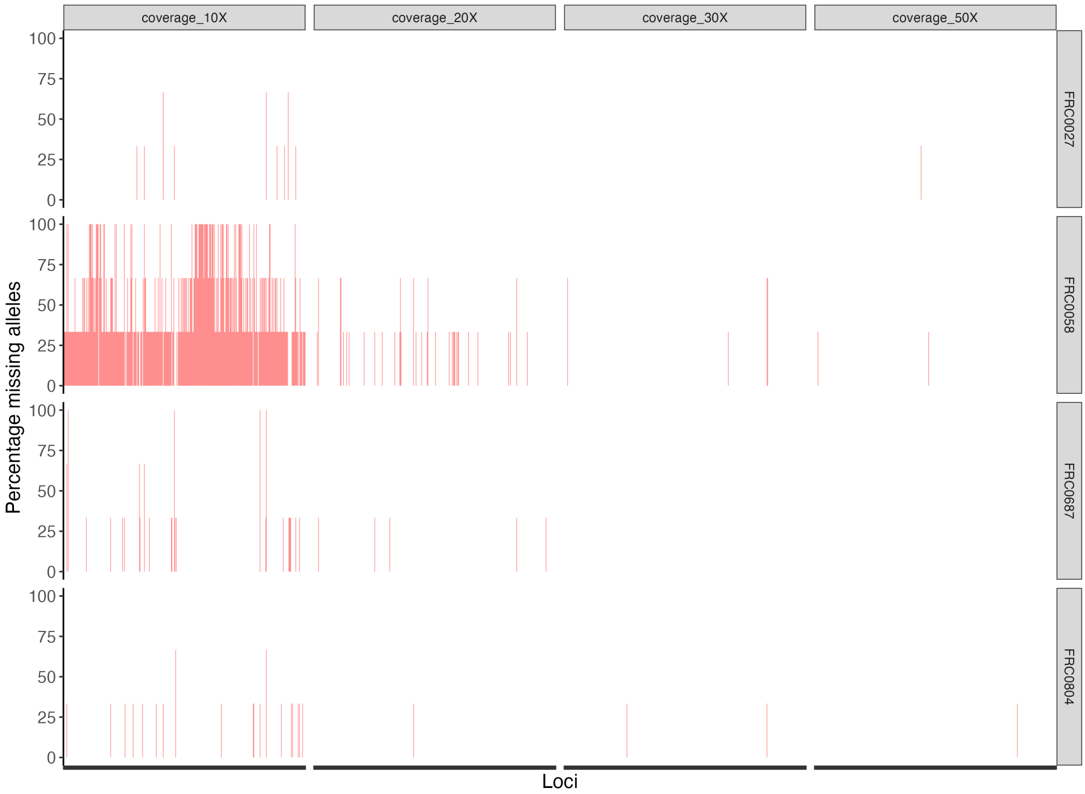

Figure S10. Allele call ability from low coverage depths for four *C. ulcerans* genomes (FRC0027, FRC0058, FRC0687, FRC0804). Genome assemblies built from simulated low coverage depth data (10×, 20×, 30×, 50×, 80×, 100×; in triplicate for each genome and each coverage) were tagged for existing alleles of the cgMLST scheme, to identify loci with non-reproducible allele calls at low assembly coverage depths. All alleles were called with no mismatch at 80× and 100×, whereas nine unique loci showed incorrect allele calls at 30× and 50×. These were discarded from the scheme, for a final set of 1,628 loci of the *C. ulcerans* cgMLST scheme.

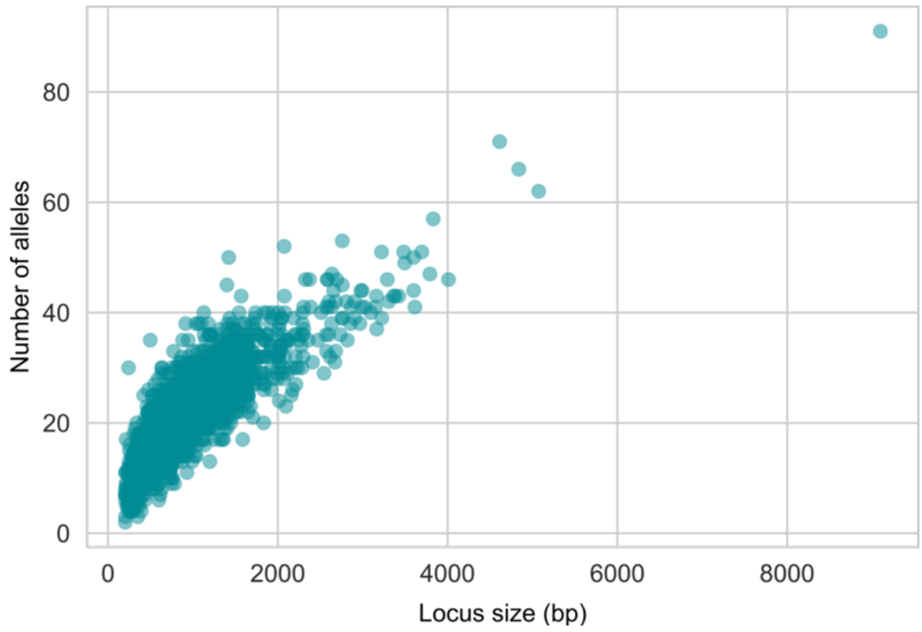

Figure S11. Scatterplot showing the relationship between the locus size and the number of alleles corresponding to that locus in the cgMLST scheme of *C. ulcerans*. The longest locus was atypically long (9,099 bp), coding for a type I polyketide synthase, and had the highest number of alleles (as expected from the positive correlation between locus size and allele number).

Supplementary table

Table S1. Case clusters of *Corynebacterium ulcerans* included in this study.

Cluster	Country	Years	no. strains	ST	SL	CG	*tox* allele	Case cluster information
**FR_01**	France	2022	3	331	331	331	fragmented	One human and their two dogs
**FR_02**	France	2022	2	514	514	514	50	One human and their dog
**FR_03**	France	2022	2	332	331	332	50	Two rats; part of FR_cryptic_cluster_17 (homeless person)
**FR_04**	France	2022	2	331	331	331	34	One human and their dog
**FR_05**	France	2021-22	109	325	325	325	49	Dog group
**FR_06**	France	2021-23	19	325	325	583	33	Cat group
**ES_01**	Spain	2019	4	514	514	514	63, fragmented	One human, their two cats and one dog
**DE_01**	Germany	2012	2	326	331	326	50	One human and their cat; NTTB isolates
**DE_02**	Germany	2007	2	326	331	10000	34	One human and a contact pig
**DE_03**	Germany	2012	2	325	325	583	33	One human and their dog; NTTB isolates
**DE_04**	Germany	2017	2	325	325	583	33	One human and a cat; NTTB isolates
**DE_05**	Germany	2010	3	331	331	10002	50	One human and two cats; cats isolates are NTTB
**UK_01**	UK	2021	2	787	428	787	43	One human and their dog
**BE_A**	Belgium	2012	1	328	331	328	50	One human in contact with cats; part of BE_cryptic_03
**BE_B**	Belgium	2013	5	332	331	332	50	One human, their three cats and one dog
**BE_C**	Belgium	2016	2	332	331	332	50	One human patient and their nurse (asymptomatic carrier)
**BE_D**	Belgium	2019	1	331	331	331	51	One human case
**BE_E**	Belgium	2020	1	332	331	332	50	One human case
**BE_G**	Belgium	2020	2	331	331	331	50	One human and their dog; part of BE_cryptic_cluster_02
**BE_K**	Belgium	2022	1	332-1LV	331	332	50	One cat

Supplementary References

[1] Maiden, M. C. J. et al. Multilocus sequence typing: a portable approach to the identification of clones within populations of pathogenic microorganisms. *Proc Natl Acad Sci U S A* **95**, 3140-3145 (1998).

[2] Jolley, K. A. & Maiden, M. C. J. BIGSdb: scalable analysis of bacterial genome variation at the population level. *BMC Bioinformatics* **11**, 595 (2010).
