# Supplementary material for: Microevolution and genomic epidemiology of the diphtheria-causing zoonotic pathogen Corynebacterium ulcerans
Source: Nat Commun. 2025 May 24;16:4843. doi: 10.1038/s41467-025-60065-0 (PMC12103533; doi:10.1038/s41467-025-60065-0)
Supplement: Supplementary file 3 — Description of Additional Supplementary Files [file 41467_2025_60065_MOESM3_ESM.pdf]

Description of Additional Supplementary Files

File Name: Supplementary Data 1
Description: *Corynebacterium ulcerans* isolates used in this study (n=582) alongside genome accession numbers, metadata, results of genomic analyses (e.g., MLST, cgMLST, toxin gene) and of laboratory analyses (e.g., PCR for diphtheria toxin, Elek test results, antimicrobial susceptibility testing, if available)
