# Supplementary material for: Microevolution and genomic epidemiology of the diphtheria-causing zoonotic pathogen Corynebacterium ulcerans
Source: Nat Commun. 2025 May 24;16:4843. doi: 10.1038/s41467-025-60065-0 (PMC12103533; doi:10.1038/s41467-025-60065-0)
Supplement: Supplementary file 5 — Reporting Summary [file 41467_2025_60065_MOESM5_ESM.pdf]

nature portfolio

Corresponding author(s): Sylvain Brisse

Last updated by author(s): Feb 21, 2025



# Reporting Summary

Nature Portfolio wishes to improve the reproducibility of the work that we publish. This form provides structure for consistency and transparency in reporting. For further information on Nature Portfolio policies, see our Editorial Policies and the Editorial Policy Checklist.

## Statistics

For all statistical analyses, confirm that the following items are present in the figure legend, table legend, main text, or Methods section.

| n/a | Confirmed | |
|---|---|---|
| ☒ | ☐ | The exact sample size (*n*) for each experimental group/condition, given as a discrete number and unit of measurement |
| ☒ | ☐ | A statement on whether measurements were taken from distinct samples or whether the same sample was measured repeatedly |
| ☒ | ☐ | The statistical test(s) used AND whether they are one- or two-sided<br>*Only common tests should be described solely by name; describe more complex techniques in the Methods section.* |
| ☒ | ☐ | A description of all covariates tested |
| ☒ | ☐ | A description of any assumptions or corrections, such as tests of normality and adjustment for multiple comparisons |
| ☒ | ☐ | A full description of the statistical parameters including central tendency (e.g. means) or other basic estimates (e.g. regression coefficient) AND variation (e.g. standard deviation) or associated estimates of uncertainty (e.g. confidence intervals) |
| ☒ | ☐ | For null hypothesis testing, the test statistic (e.g. *F*, *t*, *r*) with confidence intervals, effect sizes, degrees of freedom and *P* value noted<br>*Give P values as exact values whenever suitable.* |
| ☒ | ☐ | For Bayesian analysis, information on the choice of priors and Markov chain Monte Carlo settings |
| ☒ | ☐ | For hierarchical and complex designs, identification of the appropriate level for tests and full reporting of outcomes |
| ☒ | ☐ | Estimates of effect sizes (e.g. Cohen's *d*, Pearson's *r*), indicating how they were calculated |

*Our web collection on statistics for biologists contains articles on many of the points above.*

## Software and code

Policy information about availability of computer code

| | |
|---|---|
| Data collection | BioNumerics v7.5 was used to store isolate-associated metadata and results of phenotypic tests. BIGSdb-Pasteur (https://bigsdb.pasteur.fr/) was used to store selected metadata and results of bioinformatic analyses. |
| Data analysis | All software used to analyze the data in this study is open-source and available on-line. All software is cited in the methods section, a list follows: fq2dna v21.06, Guppy v6.4.2, Unicycler v0.4.8, diphtOscan (https://gitlab.pasteur.fr/BEBP/diphtoscan), chewBBACA v2.8.5, MSTclust (https://gitlab.pasteur.fr/GIPhy/MSTclust), blast (https://blast.ncbi.nlm.nih.gov/Blast.cgi), PHASTER (https://phaster.ca/), bwa-mem2 v2.2.1, samtools v1.18, Panaroo v1.5.047, IQ-TREE v2.0.6, MAFFT v7.526, Clustal-Omega v1.2.452, FastTree v2.1.1153, Phytools v2.1-1, IntegronFinder32 (https://github.com/gem-pasteur/Integron_Finder), BIGSdb-Pasteur (https://bigsdb.pasteur.fr/) |

For manuscripts utilizing custom algorithms or software that are central to the research but not yet described in published literature, software must be made available to editors and reviewers. We strongly encourage code deposition in a community repository (e.g. GitHub). See the Nature Portfolio guidelines for submitting code & software for further information.

## Data

Policy information about availability of data

All manuscripts must include a data availability statement. This statement should provide the following information, where applicable:

- Accession codes, unique identifiers, or web links for publicly available datasets
- A description of any restrictions on data availability
- For clinical datasets or third party data, please ensure that the statement adheres to our policy

All genome sequences published in this study were made available at BIGSdb-Pasteur (project id 45 at https://bigsdb.pasteur.fr/cgi-bin/bigsdb/bigsdb.pl?db=pubmlst_diphtheria_isolates). Supplementary material includes Dataset S1 (isolate metadata for strains used in this study) and a Supplementary Information file. A project with the phylogenetic tree, metadata and results file is available within Microreact at: https://microreact.org/project/pJf35xQkcs2HUznD67W7Be-corynebacterium-ulcerans-genomic-epidemiology-2024.

## Research involving human participants, their data, or biological material

Policy information about studies with human participants or human data. See also policy information about sex, gender (identity/presentation), and sexual orientation and race, ethnicity and racism.

| | |
|---|---|
| Reporting on sex and gender | NA |
| Reporting on race, ethnicity, or other socially relevant groupings | NA |
| Population characteristics | NA |
| Recruitment | NA |
| Ethics oversight | NA |

Note that full information on the approval of the study protocol must also be provided in the manuscript.

# Field-specific reporting

Please select the one below that is the best fit for your research. If you are not sure, read the appropriate sections before making your selection.

☒ Life sciences ☐ Behavioural & social sciences ☐ Ecological, evolutionary & environmental sciences

For a reference copy of the document with all sections, see nature.com/documents/nr-reporting-summary-flat.pdf

# Life sciences study design

All studies must disclose on these points even when the disclosure is negative.

| | |
|---|---|
| Sample size | A total of 582 C. ulcerans genome sequences, and 36 C. ramonii, were selected for this study. |
| Data exclusions | NA |
| Replication | NA |
| Randomization | NA |
| Blinding | NA |

# Reporting for specific materials, systems and methods

We require information from authors about some types of materials, experimental systems and methods used in many studies. Here, indicate whether each material, system or method listed is relevant to your study. If you are not sure if a list item applies to your research, read the appropriate section before selecting a response.

## Materials & experimental systems

| n/a | Involved in the study |
| --- | --- |
| ☒ | ☐ Antibodies |
| ☒ | ☐ Eukaryotic cell lines |
| ☒ | ☐ Palaeontology and archaeology |
| ☒ | ☐ Animals and other organisms |
| ☒ | ☐ Clinical data |
| ☒ | ☐ Dual use research of concern |
| ☒ | ☐ Plants |

## Methods

| n/a | Involved in the study |
| --- | --- |
| ☒ | ☐ ChIP-seq |
| ☒ | ☐ Flow cytometry |
| ☒ | ☐ MRI-based neuroimaging |

# Plants

| | |
| --- | --- |
| Seed stocks | NA |
| Novel plant genotypes | NA |
| Authentication | NA |
